# A COX-2/sEH dual inhibitor PTUPB alleviates lipopolysaccharide-induced acute lung injury in mice by inhibiting NLRP3 inflammasome activation

**DOI:** 10.7150/thno.43108

**Published:** 2020-03-26

**Authors:** Hui-Hui Yang, Jia-Xi Duan, Shao-Kun Liu, Jian-Bing Xiong, Xin-Xin Guan, Wen-Jing Zhong, Chen-Chen Sun, Chen-Yu Zhang, Xiao-Qin Luo, Yan-Feng Zhang, Ping Chen, Bruce D. Hammock, Sung Hee Hwang, Jian-Xin Jiang, Yong Zhou, Cha-Xiang Guan

**Affiliations:** 1 Department of Physiology, Xiangya School of Medicine, Central South University, Changsha, Hunan 410078, China;; 2 Department of Pulmonary and Critical Care Medicine, the Second Xiangya Hospital, Central South University, Changsha, Hunan 410011, China;; 3 Research Unit of Respiratory Disease, Central South University, Changsha, Hunan 410011, China;; 4 Hunan Diagnosis and Treatment Center of Respiratory Disease, Central South University, Changsha, Hunan 410011, China;; 5 Department of Entomology and Nematology and UC Davis Comprehensive Cancer Center, University of California, Davis, One Shields Avenue, Davis, CA 95616, USA;; 6 State Key Laboratory of Trauma, Burns, and Combined Injury, Army Medical University, Chongqing, 400038, China.

**Keywords:** acute lung injury, COX-2/sEH dual inhibitor, NLRP3 inflammasome, oxidative stress

## Abstract

**Rationale**: Dysregulation of arachidonic acid (ARA) metabolism results in inflammation; however, its role in acute lung injury (ALI) remains elusive. In this study, we addressed the role of dysregulated ARA metabolism in cytochromes P450 (CYPs) /cyclooxygenase-2 (COX-2) pathways in the pathogenesis of lipopolysaccharide (LPS)-induced ALI in mice.

**Methods**: The metabolism of CYPs/COX-2-derived ARA in the lungs of LPS-induced ALI was investigated in C57BL/6 mice. The COX-2/sEH dual inhibitor PTUPB was used to establish the function of CYPs/COX-2 dysregulation in ALI. Primary murine macrophages were used to evaluate the underlying mechanism of PTUPB involved in the activation of NLRP3 inflammasome *in vitro*.

**Results**: Dysregulation of CYPs/COX-2 metabolism of ARA occurred in the lungs and in primary macrophages under the LPS challenge. Decrease mRNA expression of *Cyp2j9, Cyp2j6,* and *Cyp2j5* was observed, which metabolize ARA into epoxyeicosatrienoic acids (EETs). The expressions of COX-2 and soluble epoxide hydrolase (sEH), on the other hand, was significantly upregulated. Pre-treatment with the dual COX-2 and sEH inhibitor, PTUPB, attenuated the pathological injury of lung tissues and reduced the infiltration of inflammatory cells. Furthermore, PTUPB decreased the pro-inflammatory factors, oxidative stress, and activation of NACHT, LRR, and PYD domains-containing protein 3 (NLRP3) inflammasome in LPS-induced ALI mice. PTUPB pre-treatment remarkably reduced the activation of macrophages and NLRP3 inflammasome* in vitro*. Significantly, both preventive and therapeutic treatment with PTUPB improved the survival rate of mice receiving a lethal dose of LPS.

**Conclusion**: The dysregulation of CYPs/COX-2 metabolized ARA contributes to the uncontrolled inflammatory response in ALI. The dual COX-2 and sEH inhibitor PTUPB exerts anti-inflammatory effects in treating ALI by inhibiting the NLRP3 inflammasome activation.

## Introduction

Acute lung injury (ALI) and acute respiratory distress syndrome (ARDS) are life-threatening diseases characterized by uncontrolled inflammatory responses, elevated penetrability of the alveolar- capillary barrier, and pulmonary edema 1, 2. Inflammatory response-activated macrophages, and neutrophils infiltrate into the lung. Cytokines are released by infiltrating cells activating local pro-inflammatory networks and reactive oxygen species (ROS) 3, 4. Also, NACHT, LRR and PYD domain-containing protein 3 (NLRP3) inflammasome plays a pivotal role in the pathogenesis of ALI 5, 6, and could cause irrevocable damage to lung epithelium and endothelial cells 7, 8. As a major constituent of the outer membrane of gram-negative bacteria, lipopolysaccharide (LPS) induces a diverse spectrum of infections, including life-threatening pneumonia and septicemia. The signaling also actives the downstream nuclear factor kappa-B (NF-κB) and augments inflammatory mediators 9. Hence, LPS has emerged as a clinically relevant model for ALI 10.

The therapeutic approach to inflammatory diseases has mainly focused on targeting various fatty acids and lipids, such as arachidonic acid (ARA), which could be metabolized into eicosanoids. The cyclooxygenase (COX), lipoxygenase (LOX), and cytochrome p450 (CYP) are the three primary pathways for the production of eicosanoids 11. Collective evidence suggests that the epoxyeicosatrienoic acids (EETs) derived from the CYP pathway confer anti-inflammatory effects 12, 13. However, EETs are rapidly metabolized into the corresponding dihydroxyeicosatrienoic acids (DHETs) and diols mainly by the soluble epoxide hydrolase (sEH) 14. The sEH, a multifunctional protein encoded by the *EPHX2* gene, is expressed in numerous tissues, including the lungs 15. Our laboratory as well as other researchers previously reported that inhibition of sEH or gene knockout exerts protective effects against ALI 16, 17 and pulmonary fibrosis 18. COX acts as the critical enzyme converting ARA to prostaglandins (PGs), which are generally very low in physiological conditions but increase in acute inflammation 19. COX-2 accounts for the increased production of PGs during inflammation and immune responses 20. The expression of COX-2 significantly increases during the development of ALI, and suppressing COX-2 attenuates LPS-induced ALI 21.

Inhibition of a specific biosynthetic ARA pathway may alter the metabolic flux resulting in fatal side effects 22. sEH inhibitors and EETs can increase the expression of COX-2 23, 24. Therefore, it is imperative to develop novel anti-inflammatory strategies with a dual mechanism, which prevent the release of pro-inflammatory PGs and enhance the concentration of EETs. PTUPB (4-(5-phenyl-3-{3-[3-(4-trifluoromethylphenyl)-ureido]-propyl}-pyrazol-1-yl)-benzenesulfonamide), is a novel dual COX-2 and sEH inhibitor, which was developed in our previous study 25. It reduces the levels of COX-dependent PGs and increases the CYPs-dependent metabolites 26, indicating the suppression of both COX-2 and sEH pathways. Recently, we reported that PTUPB suppresses the growth of glioblastoma 27, reduces kidney injury and sepsis 28, 29, and attenuates pulmonary fibrosis 30. However, it is not clear whether dual inhibition of COX-2 and sEH exerts any protective effect against ALI. In the present study, we demonstrated dysregulation of CYPs /COX-2 during ALI and effective attenuation of ALI by dual inhibition of COX-2 and sEH with PTUPB.

## Materials and Methods

### Animals

Adult (6-8 weeks, 18-20 g) male C57BL/6 mice were purchased from Hunan SJA Laboratory Animal Co., Ltd (Hunan, China). All mice were kept in a controlled environment with 24-26 °C, 50%-60% humidity, and 12 h cycle of night and day. Experimental use of mice in the present study was performed according to the guidelines of the National Institutes of Health for live animals.

### Animal treatment

Mice were randomly divided into the control, PTUPB, ALI, and ALI + PTUPB groups. ALI was induced as described in our previous study 16. Briefly, ALI was induced by intratracheal injection of LPS (5 mg/kg, from *Escherichia coli* O111: B4, Sigma-Aldrich, USA) dissolved in 50 μL sterile saline. Mice in the control and PTUPB groups received 50 μL sterile saline intratracheally. Mice in the PTUPB and PTUPB+ALI group were subcutaneously injected with PTUPB (5 mg/kg) dissolved in PEG400 1 h prior to the intratracheal injection. PEG400 was subcutaneously injected for the control and ALI groups. Mice were sacrificed 12 h after the LPS injection. A lethal dose of LPS (25 mg/kg) was injected into the trachea for survival study. PTUPB (5 mg/kg, subcutaneous) or PEG400 was administered to mice 1 h before or 6 h after the LPS injection. After the LPS injection, the survival rate was tracked every 6 h. All surgical procedures were performed under anesthesia.

### Pulmonary function measurement

The testing system of the Buxco pulmonary function (Buxco, Sharon, Connecticut, CT, USA) was employed to detect the pulmonary function of mice, including airway resistance, lung compliance, and pulmonary ventilation, which was described in our previous study 31. The pulmonary function measurement was carried out in anesthetized mice.

### Hematoxylin-Eosin (H&E) staining and inflammatory injury score analysis

Paraffin-embedded left lungs were sliced in 3-μm thickness and then stained with H&E to observe morphologic changes. Lung injury was measured as previously described 32. Briefly, the severity of the injury was graded from 0 to 4, according to five independent variables: hemorrhage, neutrophils in the alveolar space, hyaline membranes, pertinacious debris filling the airspaces, and septal thickening. The score of 0 means no damage; l, <25% damage; 2, 25 to 50% damage; 3, 50 to 75% damage and 4, > 75% damage. The inflammation score was measured independently by three pathologists blinded to the experiment. The scores from all three were averaged to give a final score.

### Bronchoalveolar lavage fluid (BALF) collection and cell counts

BALF was collected as previously described 33. Briefly, the lungs were lavaged with 0.8 mL ice-cold PBS (pH = 7.4) three times. The recovered fluid was centrifuged at 1500 rpm for 5 min at 4 ℃, and then the supernatant was collected and stored at -80 ℃ for subsequent experiments. The sedimented cell pellets were re-suspended in 0.5 mL PBS, then cells, including macrophages and neutrophils, were counted with a hemocytometer and Wright-Giemsa staining using a light microscope as described in our previous study 16.

### Evaluation of oxidative stress

The up-right lung lobes of mice were homogenized in PBS at a ratio of 1:10 (weight: volume). Total activity of superoxide dismutase (SOD), the level of malondialdehyde (MDA), and reactive oxygen species (ROS) in the lungs were assessed by corresponding kits following manufacturer's instructions (Cat# SOD: A001-3; MDA: A003-1; ROS: E004, Jiancheng Bioengineering Institute, Nanjing, China).

### Isolation and treatment of primary murine peritoneal macrophages

Primary murine peritoneal macrophages were isolated and cultured as outlined in our previous study 34. Cells were plated into 12-well plates (1×10^6^ cells/well) for gene and protein detection, 24-well plates (0.5×10^6^ cells/well) for immunofluorescence staining, 6-well plates (2×10^6^ cells/well) for ROS evaluation, and 100 mm culture dishes (1×10^7^ cells/dish) for immunoprecipitation (IP). To estimate the effect of PTUPB on LPS (100 ng/mL)-challenged murine macrophages, a series of concentrations of PTUPB (10, 100, 1000, and 10000 nM) were added 1 h before LPS (100 ng/mL) stimulation; cells in the control group were treated with DMSO. Six hours later, macrophages were harvested for gene detection. Twelve hours later, macrophages were harvested for protein and immunofluorescence staining detection. To evaluate the effect of PTUPB on the NLRP3 inflammasome activation, PTUPB (1000 nM) was added to the cultures 1 h ahead of the LPS treatment (100 ng/mL) for 135 min and adenosine triphosphate (ATP, 2.5 mM) treatment for another 45 min.

### Detection of lactate dehydrogenase (LDH) activity

The activity of LDH in serum, BALF, and the supernatant was detected by using the LDH Cytotoxicity Assay Kit (Cat# A020-2, Jiancheng Bioengineering Institute, Nanjing, China).

### Real-time quantitative polymerase chain reaction (RT-qPCR)

The extraction of total RNA, generation of cDNA, and RT-qPCR were achieved as described in our previous study 33. Gene expression was measured by 2^-ΔCt^, and the relative gene expression was assayed by 2^-ΔΔCt^ according to the previous study 35, 36. Primers used in this study were synthesized by Sangon Biotech (Shanghai, China) as our previous studies 30, 33, and the sequences are shown in Table 1.

### Cytokine detection

The contents of tumor necrosis factor-alpha (TNF-α), monocyte chemotactic protein 1 (MCP-1), and interleukin-1 beta (IL-1β) in BALF, serum, cell culture supernatant, or the lung tissue were measured using ELISA kits (Cat# TNF-α: 88-7324; MCP-1: 88-7391; IL-1β: 88-7013; Invitrogen, Thermo Fisher Scientific, USA). The contents were assayed by comparison of the optical density (450 nm and 570 nm) with the standard curve.

### Western blotting

Proteins from the lung tissue or macrophages were extracted and analyzed using Western blotting as described in our previous study 37. Frozen lung tissue was homogenized and lysed in RIPA buffer (Solarbio, Beijing, China) containing a protease inhibitor (Roche, Mannheim, Germany). The protein concentrations were measured with Pierce™ BCA Protein Assay Kit (Cat# 23225, Thermo Fisher Scientific, Grand Island, NY, USA). Samples mixed with the loading buffer were separated on an SDS-PAGE gel. After transferring to polyvinylidene fluoride membranes (Millipore, Bedford, MA) by electrotransfer, the membranes were blocked with 5% BSA or non-fat milk at room temperature for 1.5 h, incubated with primary antibodies at 4 °C overnight, and then incubated with peroxidase-conjugated secondary antibodies at room temperature for 1 h. The image of protein brands was captured by a gel imaging system. The relative band intensity was measured using the Image Lab Analyzer software (Bio-Rad, Hercules, CA, USA). The antibodies used in the study are shown in Table 2.

### IP assay

For the detection of the interaction between NLRP3 and ASC (apoptosis-associated speck-like protein containing a CARD), primary murine peritoneal macrophages were washed with PBS (pH = 7.4) three times, collected and lysed with lysis buffer including complete protease inhibitor PMSF (Solarbio, P0100) and Protease Inhibitor Cocktail (MCE, HY-K0010) on ice for 35 min, then centrifuged at 12,000 rpm for 10 min at 4 °C. The supernatant (100 μL) was transferred to another cold tube and incubated with 55 μL Dynabeads™ Protein G (Invitrogen, 10004D) at 4 °C for 3 h, centrifuged at 500 g for 10 min at 4 °C. Subsequently, the Dynabeads were separated with The DynaMag™-2 (Invitrogen, 12321D). The supernatant (80 μL) was collected and anti-ASC (1:100; CST, 67824S) antibody was added at 4 °C overnight. Then, 30 μL Dynabeads™ Protein G was added to the supernatant and soaked for another 3 h on ice. The beads were washed with the lysis buffer three times and the Dynabeads were separated with The DynaMag™-2. The immunoprecipitated proteins were analyzed by Western blotting.

### Statistical analysis

All experiments were independently repeated three times. Results were shown as the mean ± SD values. All data were analyzed with SPSS 22.0 (IBM, Chicago, IL) or GraphPad Prism 7 software (San Diego, CA, USA). A *P*-value of < 0.05 was regarded as statistically significant. Statistical comparisons between two groups were determined by unpaired *t*-test. Differences among multiple groups were determined by ANOVA, followed by Bonferroni correction for multiple comparison testing. The data that were not normally distributed were analyzed using nonparametric statistical analysis. The survival rate was assayed by the log-rank test.

## Results

### Dysregulation of CYPs /COX-2 metabolism of ARA occurs in the lungs and macrophages under the LPS challenge

**Figure 1:**
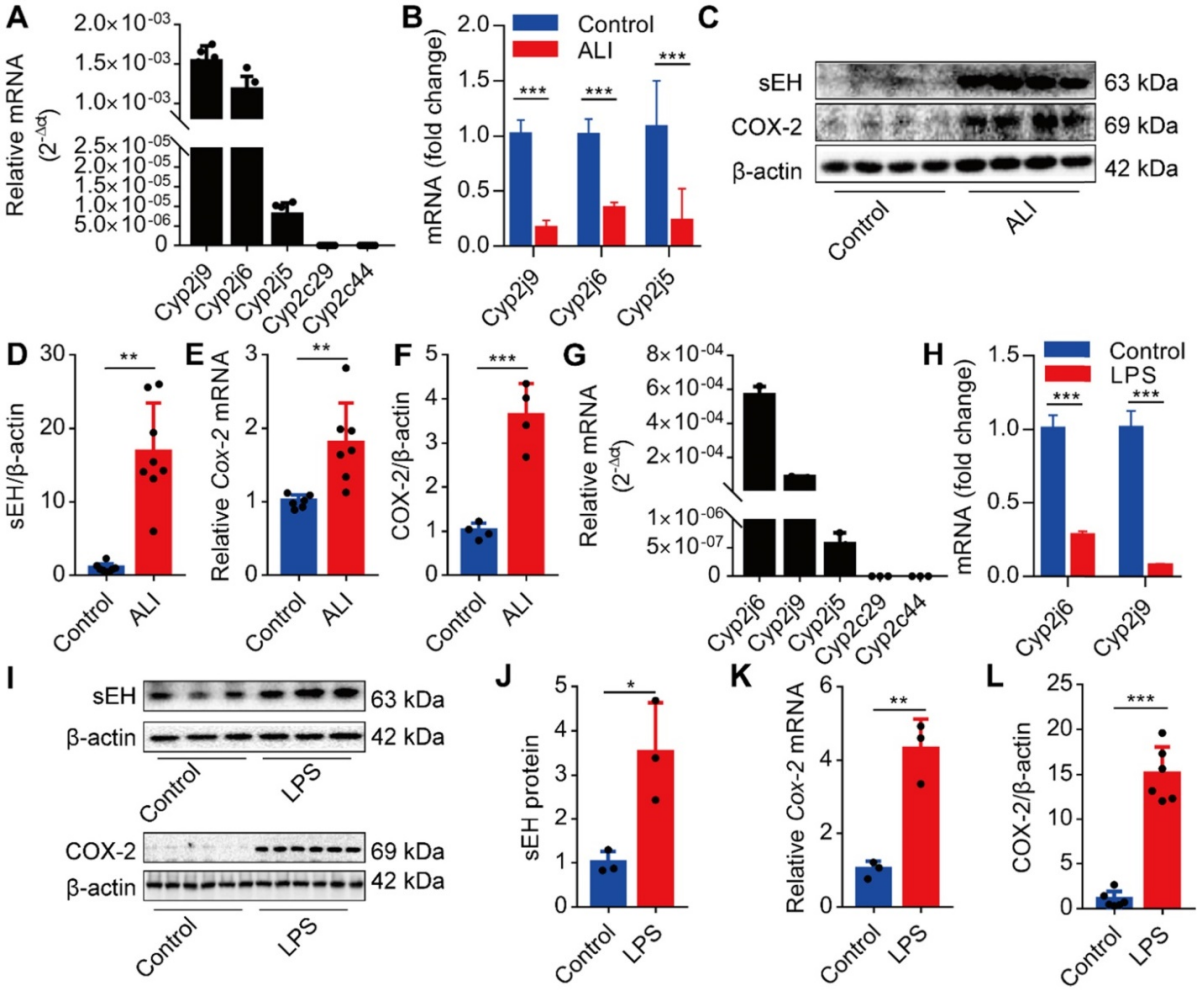
Dysregulation of CYPs/COX-2 metabolism of ARA occurs in the lungs and macrophages under the LPS challenge. *Cyp2j9* was the most abundant CYP isoform expressed in the lungs, whereas* Cyp2c29* and *Cyp2c44* mRNA were undetectable (A, *n* = 6). *Cyp2j9*, *Cyp2j6,* and *Cyp2j5* mRNAs were robustly decreased at 12 h after LPS administration (5 mg/kg,* i.t.*) in the lungs (B, *n* = 6). Western blotting and RT-qPCR results showing increased sEH and COX-2 proteins, and *Cox-2* mRNA at 12 h after LPS administration. (C-F, *n* = 4-8). *Cyp2j6* was the most abundant CYP isoform expressed in primary murine macrophages after treatment with LPS (100 ng/mL), whereas* Cyp2c29* and *Cyp2c44* mRNA were undetectable (G, *n* = 3). *Cyp2j6* and *Cyp2j9* mRNA were remarkably decreased at 6 h after LPS administration (H, *n* = 3). Expression of sEH and COX-2 protein and *Cox-2* mRNA were detected by Western blotting and RT-qPCR (I-L, *n* = 3). Data are expressed as the mean ± SD. * *P <* 0.05, ** *P <* 0.01, and *** *P <* 0.001.

We first investigated whether the CYPs/COX-2 metabolism-derived ARA was altered during ALI. We analyzed the expression of *Cyp2j9*, *Cyp2j6*, *Cyp2j5*, *Cyp2c29*, and *Cyp2c44* in the lungs of mice and found that *Cyp2j9* and *Cyp2j6* were expressed in the lungs, whereas *Cyp2c29* and *Cyp2c44* mRNAs were undetectable (Figure 1A). Under LPS stimulation, the expression of *Cyp2j9*, *Cyp2j6*, and *Cyp2j5* mRNAs in the lungs was significantly abated (Figure 1B), while the expression of sEH and COX-2 was considerably increased (Figure 1C-F). Since macrophages have pivotal roles in the initiation of innate immune response at the early stage of ALI, we used primary murine macrophages to evaluate the expression pattern of CYPs/COX-2 metabolism of ARA *in vitro*. The results were consistent with those of the lungs *in vivo* (Figure 1G-L). Collectively, these data indicate that the dysregulation of CYPs/COX-2 metabolism of ARA occurs in the lungs and macrophages under the LPS challenge.

### PTUPB attenuates the pathological lung injury and rescues the respiratory function of mice treated with LPS

**Figure 2:**
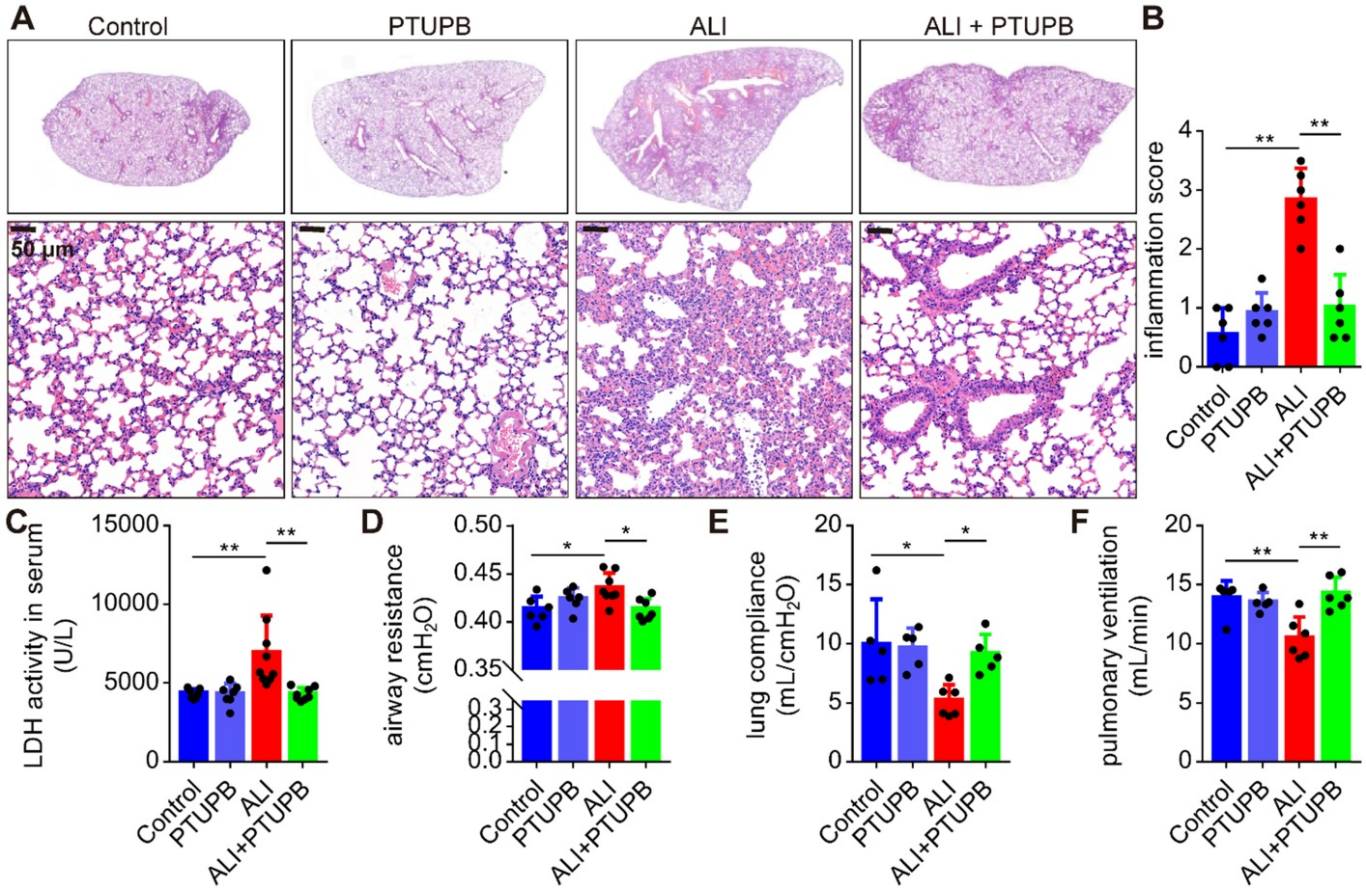
PTUPB attenuates the pathological lung injury and rescues the respiratory function of ALI mice. C57BL/6 mice were subcutaneously injected with PTUPB 1 h before the LPS administration. Twelve hours after LPS injection (5 mg/kg, *i.t.*), lung histopathology was performed with H&E staining (A). Inflammation score was measured independently by three pathologists blinded to the experiment (B, *n* = 6). Activity of LDH in the serum was assayed (C, *n* = 7-8). Respiratory function was detected by Buxco, including airway resistance (D, *n* = 5-8), lung compliance (E, *n* = 5-8), and pulmonary ventilation (F, *n* = 5-8). Data are mean ± SD. * *P* < 0.05, ** *P* < 0.01, and *** *P* < 0.001.

We used PTUPB for the dual inhibition of COX-2 and sEH to establish the function of CYPs/COX-2 dysregulation in ALI. PTUPB reduced the expression of sEH and COX-2 (Figure S1A-C), but had no effect on the expression of* Cyp2j9* and *Cyp2j6* (Figure S1D- E), as well as *Alox5/12/15* (encoding 5/12/15-LOX, Figure S1F-H). Following LPS stimulation, there was an increase in the thickness of the alveolar wall, interstitial infiltrated inflammatory cells, and the collapse of the alveoli in the lungs of mice (Figure 2A). Remarkably, PTUPB pre-treatment alleviated these pathological changes (Figure 2A-B) and pronounced reduced the LDH activity in the serum (Figure 2C). Additionally, we evaluated the effects of PTUPB on the respiratory function of ALI mice. The results showed that PTUPB pre-treatment significantly reduced airway resistance (Figure 2D), and increased lung compliance and pulmonary ventilation of mice with ALI induced by LPS (Figure 2E-F). These results demonstrate that PTUPB attenuates pathological injury of the lung tissue and enhances the respiratory function of LPS-treated ALI mice.

### PTUPB reverses the infiltration of inflammatory cells and oxidative stress in ALI mice treated with LPS

**Figure 3:**
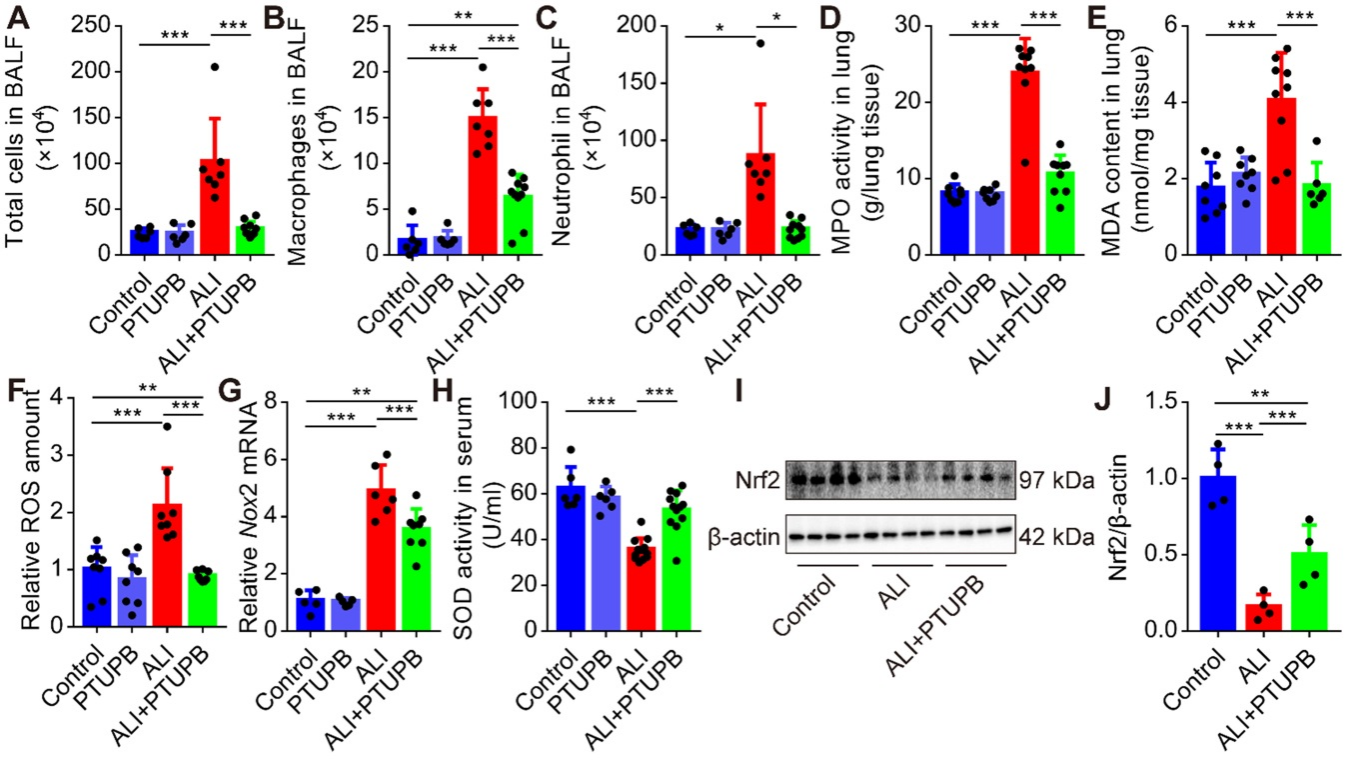
PTUPB reverses the infiltration of inflammatory cells and oxidative stress in ALI mice. C57BL/6 mice received LPS injection (5 mg/kg, *i.t.*) with or without PTUPB pre-treatment (5 mg/kg) for 1 h. Twelve hours after the LPS injection, the total cells (A), macrophages (B), and neutrophils (C) in the BALF were counted (*n* = 6-10). MPO activity was detected to quantify the infiltration of neutrophils in the lungs (D, *n* = 7-10). MDA and total ROS in the lungs were detected 12 h after the LPS administration (E-F, *n* = 6-9). mRNA expression of *Nox2* in the lungs was detected by RT-qPCR (G, *n* = 5-8). Total SOD activity in serum was detected 12 h after the LPS administration (H, *n* = 6-10). Protein expression of Nrf2 in the lungs was assayed by Western blotting (I-J, *n* = 4-8). Data are mean ± SD. * *P* < 0.05, ** *P* < 0.01, and *** *P* < 0.001.

Next, we found that pre-treatment PTUPB remarkably reduced the number of total cells (Figure 3A), macrophages (Figure 3B), and neutrophils (Figure 3C) in the BALF, as well as MPO activity in the lungs of ALI mice (Figure 3D). Immunofluorescence results confirmed the inhibitory effects of PTUPB on the accumulation of neutrophil and macrophages (Figure S2). One of the characteristic features of ALI is oxidative stress. Pre-treatment with PTUPB reduced the MDA content and total ROS in the lungs of ALI mice (Figure 3E-F). Furthermore, NADPH oxidases (NOXs) are important sources for the regulated generation of ROS 38 and our results demonstrated that PTUPB suppressed the expression of *Nox2* mRNA (Figure 3G). PTUPB pre-treatment also restored the activity of the anti-oxidative enzyme SOD (Figure 3H) in serum, and partially restored the protein expression of nuclear factor-erythroid-2- related-factor-2 (Nrf2) in the lungs, which were both reduced in ALI mice (Figure 3I-J). Collectively, these data reveal that the dual inhibition of COX-2/sEH reverses the accumulation of the inflammatory cells and oxidative stress.

### PTUPB reverses the augment of pro-inflammatory factors in the lungs of ALI mice

**Figure 4:**
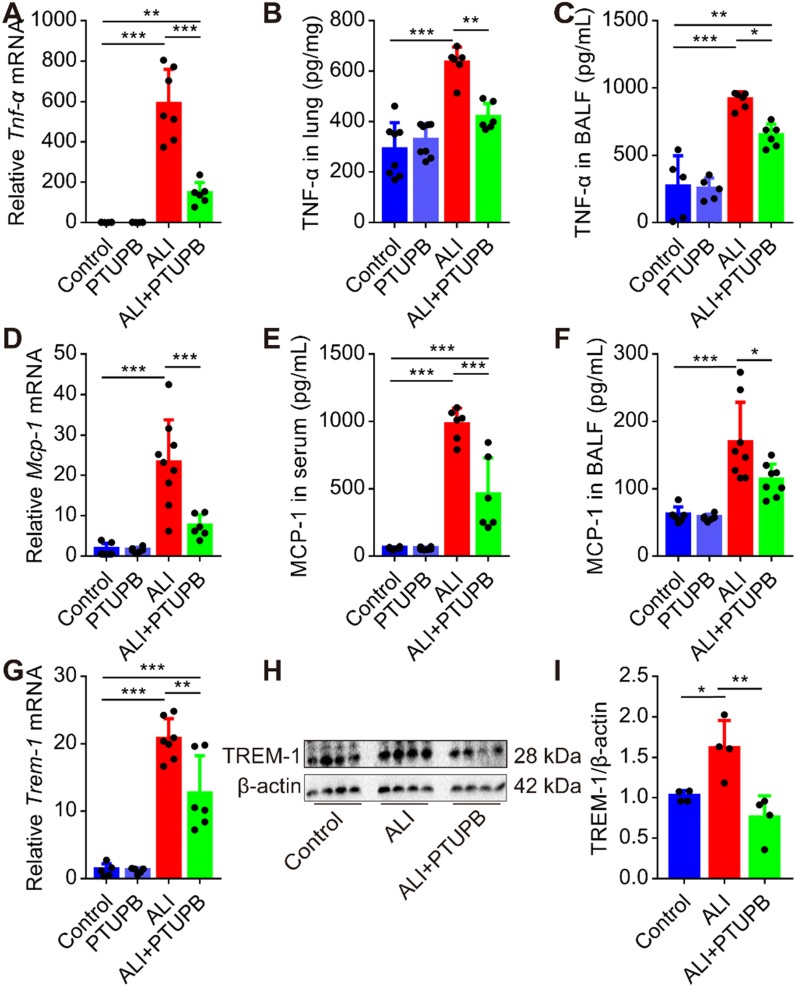
PTUPB attenuates the production of pro-inflammatory factors in the lungs of ALI mice. C57BL/6 mice received LPS injection (5 mg/kg,* i.t.*) with or without PTUPB pre-treatment (5 mg/kg) for 1 h. Twelve hours after the LPS administration, mRNA expression of *Tnf-α* (A, *n* = 6-7), *Mcp-1* (D, *n* = 6-7), and* Trem-1* (G, *n* = 5-7) in the lungs was detected by RT-qPCR. Protein content of TNF-α in lung tissue (B, *n* = 6-8) and BALF (C, *n* = 6-8), MCP-1 in serum (E, *n* = 5-8) and BALF (F, *n* = 6-8) was assayed by ELISA. Expression of TREM-1 in lung tissue was assayed by Western blotting (H-I, *n* = 4). Data are expressed as the mean ± SD. * *P* < 0.05, ** *P* < 0.01, and *** *P* < 0.001.

“Cytokine storms” is well characterized in ALI. TNF-α-targeting aptamer with antagonistic effect on TNF-α attenuates the severity of acute lung injury 39. We found that the expression of TNF-α and MCP-1 was profoundly up-regulated in the lungs of mice 12 h post LPS treatment (Figure 4A-F). PTUPB pre-treatment remarkably suppressed the increase in TNF-α and MCP-1 expression in the lungs, serum, or BALF of ALI mice (Figure 4A-F). Our pervious study indicated that the triggering receptor expressed on myeloid cells (TREM-1) acted as a pivotal inflammatory amplifier receptor in ALI 37. Here, we found that PTUPB pre-treatment also down-regulated the mRNA and protein expression of TREM-1 in the lungs of ALI mice (Figure 4G-I). Altogether, our data indicate that blockade of COX-2/sEH reduces the pro- inflammatory factors in ALI mice induced by LPS.

### PTUPB inhibits activation of NLRP3 inflammasome in the lungs of ALI mice

**Figure 5:**
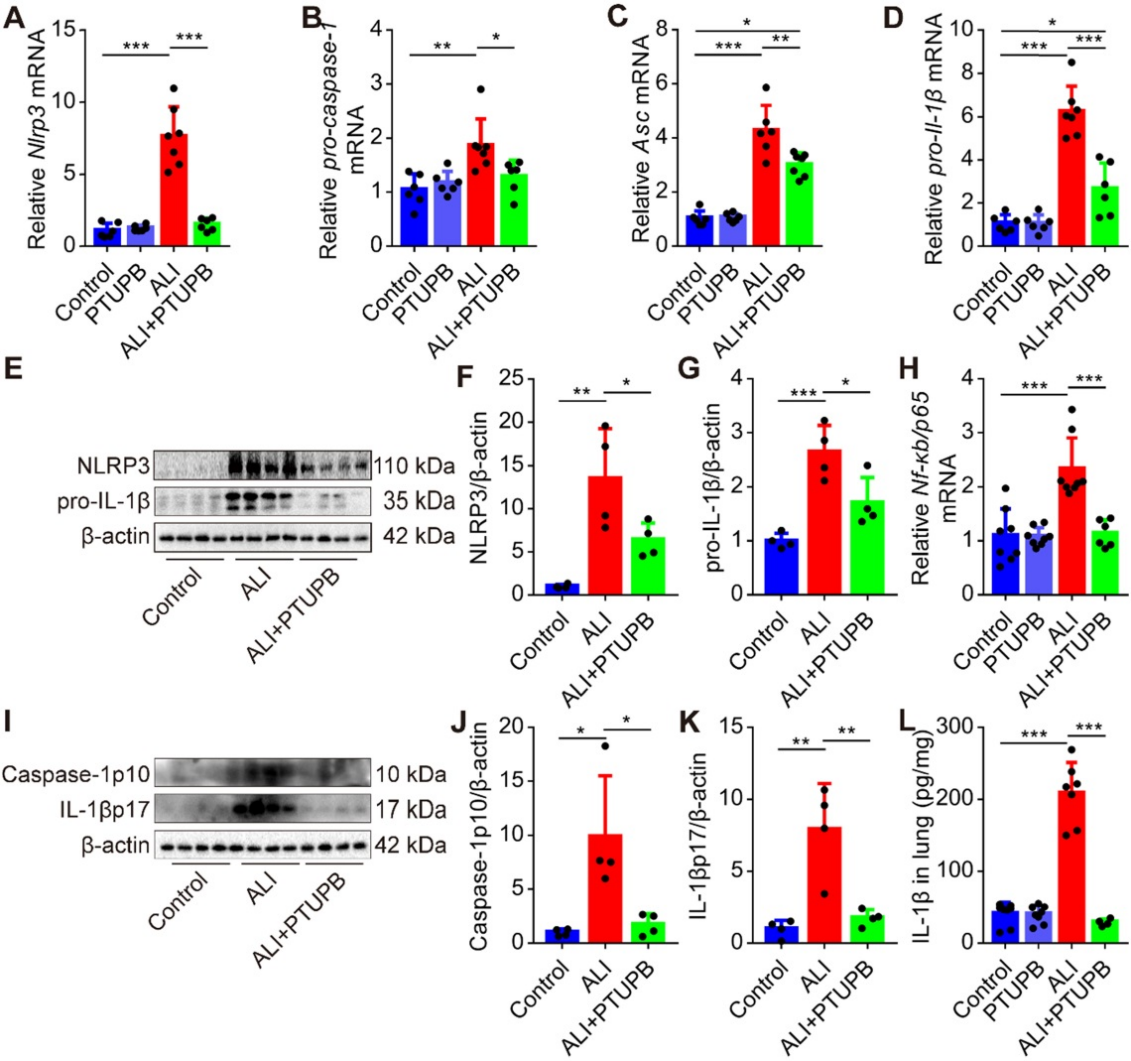
PTUPB inhibits the activation of NLRP3 inflammasome in the lung of ALI mice. C57BL/6 mice received LPS injection (5 mg/kg, *i.t.*) with or without PTUPB pre-treatment (5 mg/kg) for 1 h. mRNA expression of *Nlrp3* (A, *n* = 6-7), *pro-caspase-1* (B, *n* = 6-7), *Asc* (C, *n* = 6-7), and *pro-Il-1β* (D, *n* = 6-7) in the lungs was detected by RT-qPCR 12 h after the LPS injection. Protein expression of NLRP3 and pro-IL-1β (E-G, *n* = 4) in the lungs was detected by Western blotting. mRNA expression of *Nf-κb/p65* (H, *n* = 6-9) in the lungs was detected by RT-qPCR 12 h after the LPS injection. Protein expression of caspase-1p10 and IL-1βp17 in the lungs was detected by Western blotting (I-K, *n* = 4). Concentration of IL-1β in lung tissue was detected by ELISA (L, *n* = 5-8). Data are expressed as the mean ± SD. * *P* < 0.05, ** *P* < 0.01, and *** *P* < 0.001.

A growing body of literature has shown that the NLRP3 inflammasome plays a pivotal role in the pathogenesis of ALI 5, 6. Here, we found that blockade of COX-2/sEH by PTUPB reversed the up- regulation of components of the NLRP3 inflammasome, including* Nlrp3*, *pro-caspase-1, Asc*, and* pro-Il-1β* mRNAs in the lungs of ALI mice (Figure 5A-D). Besides, PTUPB pre-treatment also strongly reduced the expression of NLRP3 and pro-IL-1β proteins (Figure 5E-G) and NF-κB is known to be involved in the expression of these proteins. We found that PTUPB also reduced the mRNA expression of *Nf-κb/ p65* (Figure 5H). Also, caspase-1 p10 is a biomarker for the activation of NLRP3 inflammasome, which cleaves pro-IL-1β into IL-1β p17 40, 41. We found that PTUPB pre-treatment markedly blocked LPS-induced expression of caspase-1 p10 and release of IL-1β p17 (Figure 5I-L). Altogether, these data indicate that PTUPB inhibits the activation of NLRP3 inflammasome in the lungs of ALI mice.

### Prophylactic and therapeutic treatments of PTUPB significantly prevent the death of LPS-treated mice

**Figure 6:**
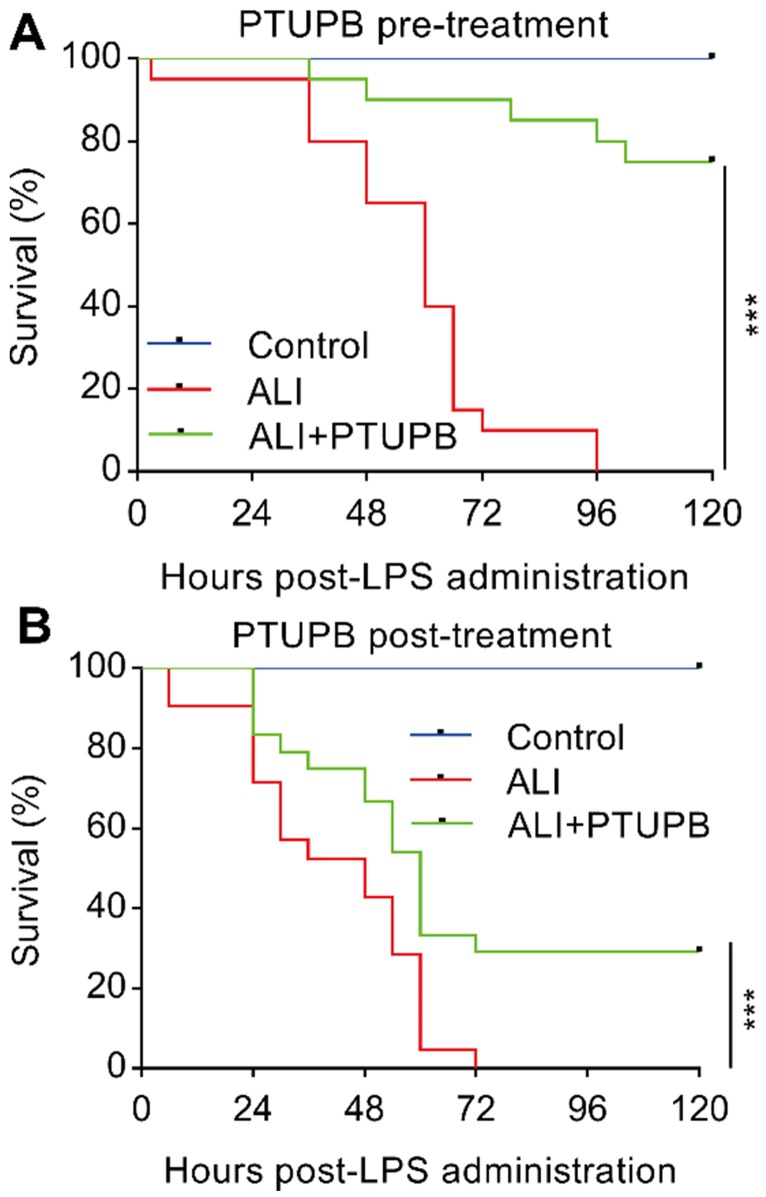
Prophylactic and therapeutic treatment of PTUPB prevents death from LPS administration in mice. PTUPB (5 mg/kg, *s.c.*) was administered to mice 1 h before (A) or 6 h after (B) induction of ALI by a lethal dose of LPS (25 mg/kg,* i.t.*). The mortality of the mice was monitored every 6 h, and the percent survival rate was expressed as a Kaplan-Meier survival curve (*n* = 20 per group). *** P < 0.001.

Pre-treatment with PTUPB (5 mg/kg) 1 h before LPS administration significantly prevented the death of mice receiving 25 mg/kg LPS (Figure 6A). We further examined the potential of therapeutic benefits of PTUPB treatment (5 mg/kg) 6 h post LPS administration. Post-treatment with PTUPB significantly prevented the death of LPS-treated mice (Figure 6B). These results indicate that blockade of COX-2/sEH by PTUPB shows prophylactic and therapeutic protection against LPS-induced ALI in mice.

### PTUPB reduces priming of NLRP3 inflammasome in primary murine macrophages

**Figure 7:**
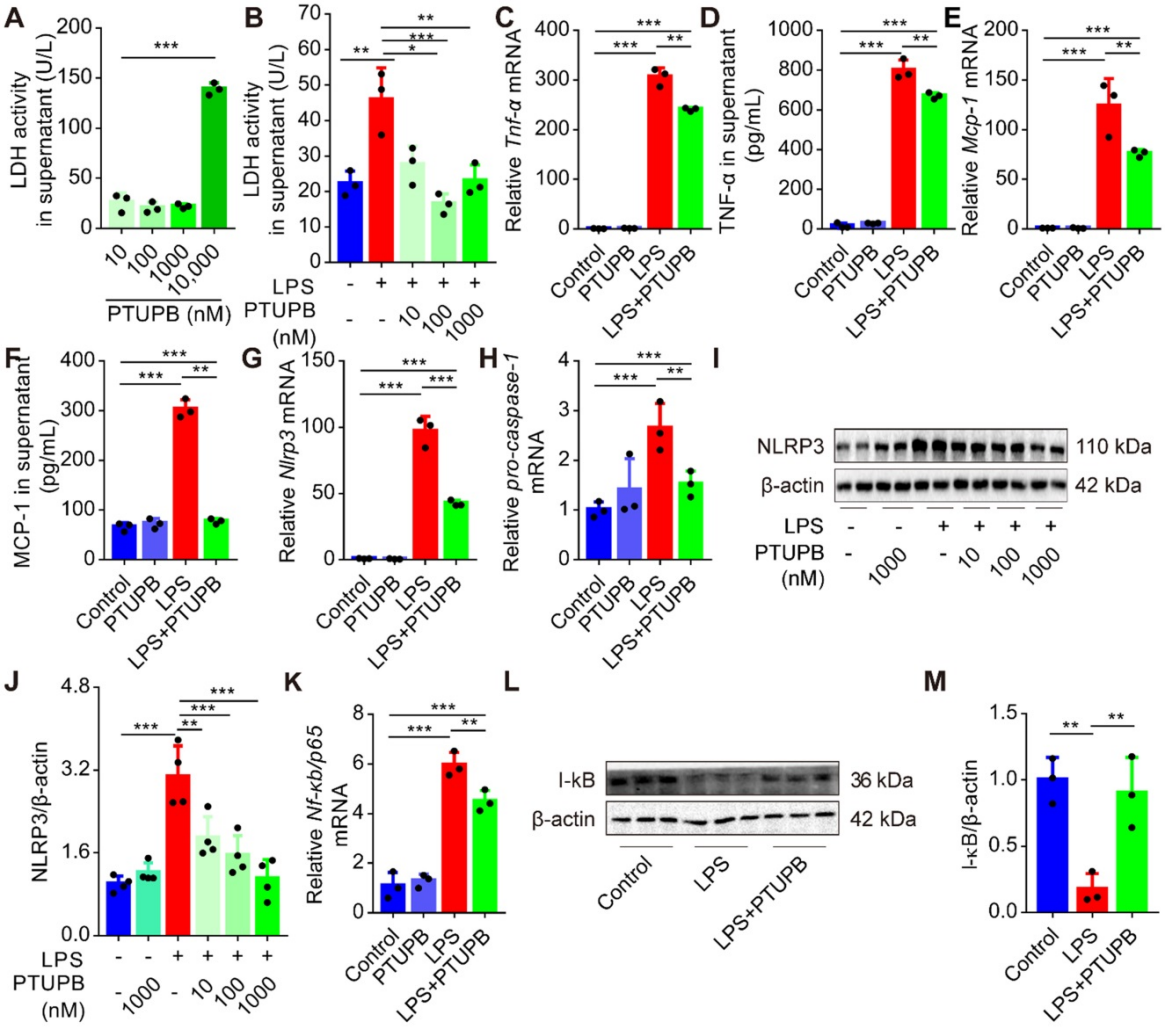
PTUPB reduces the inflammation and priming of NLRP3 inflammasome in primary murine macrophages. Primary murine macrophages were treated with serial concentrations of PTUPB (10, 100, 1000, and 10000 nM) for 6 h and the LDH activity in the supernatant of cell culture was detected (A). Macrophages were treated with LPS (100 ng/mL) for 6 h with or without PTUPB pre-treatment (10, 100, and 1000 nM) for 1 h and activity of LDH in the supernatant was detected (B). Expression of *Tnf-α* (C), *Mcp-1* (E),* Nlrp3* (G), *pro-caspase-1* (H), and *Nf-κb/p65* (K) mRNAs in macrophages was detected by RT-qPCR. Concentration of TNF-α (D) and MCP-1 (F) in the supernatant of cell culture was detected by ELISA (*n* = 3). Protein expression of NLRP3 and IκBα in macrophages was detected by Western blotting (I-J, *n* = 4; L-M, *n* = 3). Data are expressed as the mean ± SD. ** *P* < 0.01, and *** *P* < 0.001.

Treatment with clodronate-loaded liposomes that deplete macrophages significantly attenuated the lung tissue pathological injury (Figure S3A-C), and reduced the activation of NLRP3 inflammasome (Figure S3D-F). We evaluated the effects of PTUPB on the inflammatory response of primary murine macrophages and found that low-dose PTUPB (≤1000 nM) had no detectable influence on LDH release from macrophages (Figure 7A). However, PTUPB pre-treatment at 10, 100, and 1000 nM concentrations remarkably reduced the LDH activity induced by LPS (100 ng/mL) (Figure 7B). PTUPB also restored the protein expressions of COX-2 and sEH induced by LPS in macrophages (Figure S4). Furthermore, PTUPB (1000 nM) significantly reversed the expression of pro-inflammatory factors induced by LPS, including TNF-α (Figure 7C-D) and MCP-1 (Figure 7E-F). Importantly, PTUPB pre-treatment remarkably suppressed priming of NLRP3 inflammasome characterized by the inhibition of *Nlrp3* and *pro-caspase-1* mRNAs, and NLRP3 protein (Figure 7G-J). NF-κB plays a vital role in priming of NLRP3 inflammasomes 40. PTUPB pre-treatment inhibited the expression of *Nf-κb/p65* mRNA (Figure 7K), restored the expression of I-κBα protein in the lungs (Figure 7L-M), and inhibited its translocation into the cell nucleus (Figure S5). These results imply that blockade of COX-2/sEH reverses the priming of NLRP3 inflammasome in macrophages* in vitro*.

### PTUPB inhibits the activation of NLRP3 inflammasome in primary murine macrophages

**Figure 8:**
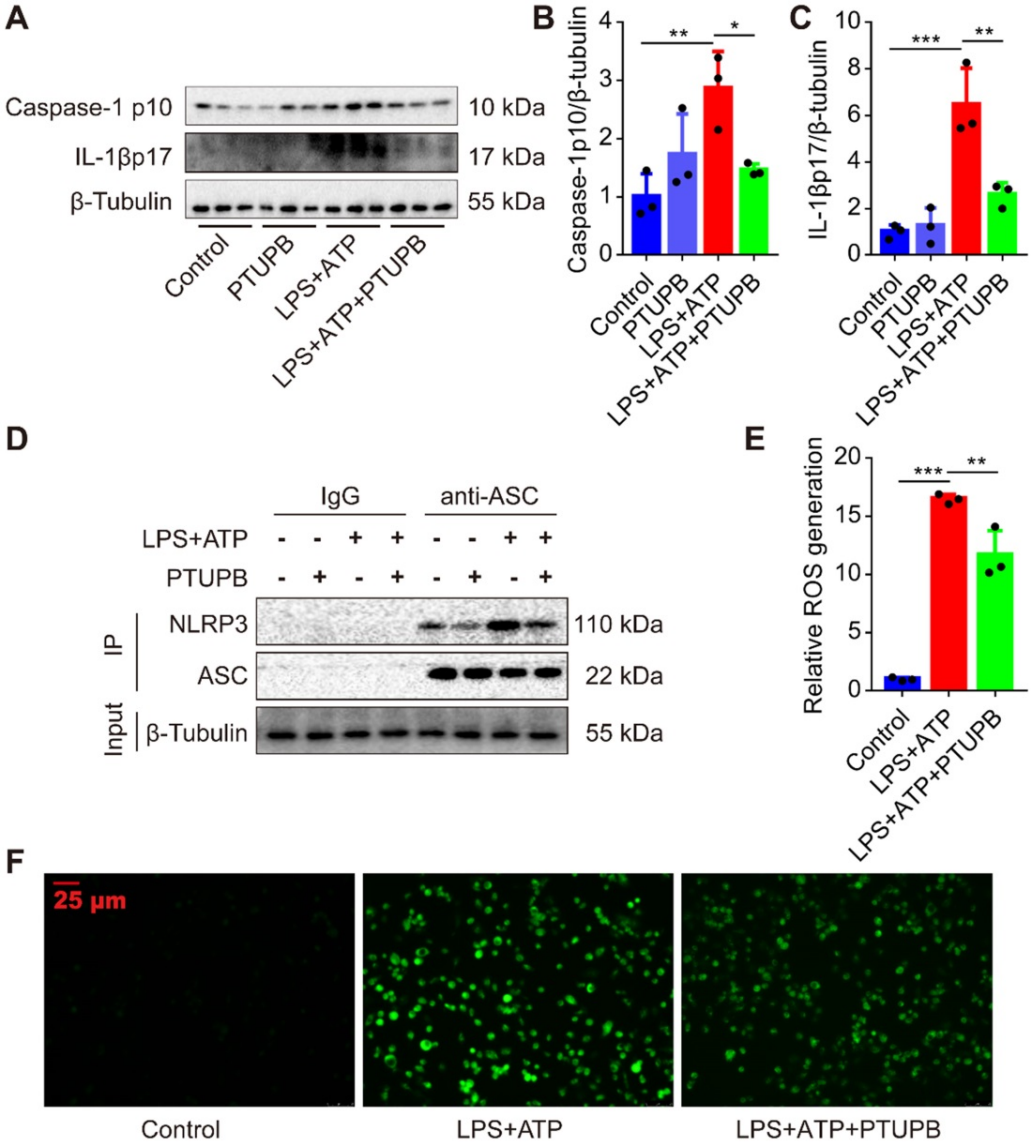
PTUPB inhibits the activation of NLRP3 inflammasome in primary murine macrophages*.* To evaluate the effects of PTUPB on the activation of NLRP3 inflammasome, PTUPB (1000 nM) was added 1 h before LPS-priming (100 ng/mL) for 135 min. Subsequently, cells were stimulated with ATP (2.5 mM) for 45 min. Protein expression of caspase-1 p10 and IL-1β p17 in macrophages was detected by Western blotting (A-C, *n* = 3). Interaction between endogenous NLRP3 and ASC was analyzed by IP (D). The ROS was analyzed by a ROS kit (E-F). Data are expressed as the mean ± SD. * *P* < 0.05, ** *P* < 0.01, and *** *P* < 0.001.

Lastly, we treated macrophages with LPS plus ATP to activate the NLRP3 inflammasome. We found that PTUPB reduced the protein expression of caspase-1 p10 and IL-1β p17 in macrophages (Figure 8A-C). NLRP3 complex formation is the key step for the activation of NLRP3 inflammasome 42. IP results showed that PTUPB inhibited the interaction of endogenous NLRP3 and ASC in macrophages (Figure 8D). ROS is one of the most important factors regulating the activation of NLRP3 inflammasome and PTUPB reversed the elevated ROS (Figure 8E-F). These results imply that blockade of COX-2/sEH reverses the activation of NLRP3 inflammasome in macrophages by suppressing ROS generation.

## Discussion

Our study demonstrated that CYPs/COX-2 dysregulation occurred during the inflammation induced by LPS both in mice and in cultured macrophages, which was evidenced by lower CYPs expression and higher COX-2 expression. Restoring the homeostasis of ARA metabolism by CYPs/COX-2 with PTUPB could alleviate lung tissue pathological injury, reduce oxidative stress and decrease the secretion of pro-inflammatory factors, and inhibit the activation of NLRP3 inflammasome in the lungs of ALI mice. We report here for the first time that dysregulated metabolism of CYPs/COX-2-derived ARA plays a vital role in LPS-induced ALI mice, and the dual inhibition of COX-2 and sEH may be an effective anti-inflammatory strategy in treating ALI.

Proper regulation of the biosynthesis of eicosanoids is critical for homeostasis. Detrimental consequences of the overproduction of COX and LOX pathways have been described in many inflammatory diseases in human beings 43, 44. For example, COX-2 is expressed in different cell types in the lungs, and its increased expression was observed in the lungs of LPS-induced ALI mice 21. Similarly, EETs exhibit anti-inflammatory actions in many diseases, such as central nervous system diseases 12, insulin resistance 45, and pulmonary diseases 16, 18, 34. However, litter is known about the dysregulated metabolism of CYPs/COX-2-derived ARA in LPS-induced ALI. This is the first report showing a decrease of CYP2J/2C and an increase of COX-2, and demonstrating the dysregulation of CYPs/COX-2 in the lungs of LPS-induced ALI mice.

A growing body of studies focuses on designing multiple ligands (DMLs) to augment drug efficacy, improve drug safety, and reduce side effects. Compared with combination drugs or therapies, DMLs have several advantages 22, 46, 47. We had previously designed PTUPB, which is a novel COX-2 and sEH dual inhibitor 25. We demonstrated that PTUPB could suppress the growth of glioblastoma, abate kidney injury, and exert a synergistic effect with cisplatin 26-28. Importantly, we did not observe any acute toxicity *in vivo* or *in vitro* 25. We also found that PTUPB had no effect on the expression of LOXs but significantly reduced the expression of COX-2 and sEH. In this study, we found that dual inhibition of COX-2 and sEH exhibited a powerful therapeutic potential for ALI.

One critical pathophysiological process in ALI/ARDS is manifested by extensive inflammation 48. Our laboratory as well as other investigators have shown that NLRP3 inflammasome plays a central role in the immense inflammation of ALI 6, 37. Recently, one study described that monounsaturated and polyunsaturated fatty acids could impede the NLRP3 inflammasome activation in metabolic diseases 49, but it was not clear whether CYPs/COX-2 were involved in the activation of NLRP3 inflammasome. In this study, our findings demonstrated that the dysregulated metabolism of CYPs/COX-2 was associated with the activation of NLRP3 inflammasome in the lungs of ALI mice. It has been reported that COX-2-mediated PGE_2_ positively regulates the activation of the NLRP3 inflammasome, and inhibition of COX-2 reduces NLRP3 inflammasome-derived IL-1β secretion and pyroptosis in macrophages 50, 51. We have found that inhibition of sEH suppressed the secretion of IL-1β in LPS-induced ALI mice 16, indicating that EETs could inhibit the activation of NLRP3 inflammasome. In hyperoxia-induced ALI mice, *Ephx2*^-/-^ mice showed decreased activation of NLRP3 inflammasome 52. These reports support our findings that dual inhibition of COX-2 and sEH with PTUPB suppresses the activation of NLRP3 inflammasome.

Oxidative stress is another indispensable characteristic of ALI. Our studies show that PTUPB attenuates the oxidative stress in the lungs of ALI mice, and enhances the antioxidative mechanism involving Nrf2 and SOD. It has been well documented that ROS is one of the most important factors regulating the activation of NLRP3 inflammasome 40. Although one study proposed that Nrf2 was necessary for the NLRP3 inflammasome activation 53, another one indicated that Nrf2 limited the NLRP3 inflammasome activation by down-regulating ROS production 54. Our findings demonstrated that PTUPB restored the expression of Nrf2 and reduced the oxidative stress in the lungs of LPS-induced ALI mice. Given these results, we infer that PTUPB inhibits the NLRP3 inflammasome activation by restoring the balance of oxidative and anti-oxidative mechanisms during ALI.

There are limitations to the present study. First, we evaluated the gene expression of specific critical enzymes, including *Cyp2j/2c*. It is possible that whether their mediators contribute to the mechanism of action of PTUPB in LPS-induced mice. Our previous study detected the ARA metabolites of the CYP, COX, and LOX pathways in the serum of LPS-treated mice by LC-MS/MS 55. Especially, the pro-inflammatory mediator PGE_2_ had the most significant alteration (~30 fold), and 12,13-DHOME, 9,10-DHOME in the sEH pathway were increased 55. We also found that PTUPB reduced the level of PGE_2_ derived from the COX-2 pathway and 12,13-DiHOME derived from the sEH pathway in PDX BL0269 tumor tissues 26. Thus, it appears that PTUPB treatment affects the profiles of ARA metabolites. And second, the reduction in the expression of sEH and COX-2 by PTUPB might be an indirect effect caused by inflammation, which up-regulates the expressions of sEH and COX-2 19, 56. PTUPB blocks the enzyme activities of sEH and COX-2, resulting in low inflammation. So, we propose that PTUPB not only blocks the enzyme activity but also indirectly reduces the enzyme expression.

In conclusion, this is the first report revealing that dysregulation of CYPs/COX-2-derived ARA metabolism contributes to lung injury in LPS-induced ALI mice. PTUPB, a dual COX-2 and sEH inhibitor, exerts an anti-inflammatory response and protects mice against LPS-induced ALI. Therefore, CYPs/ COX-2 dysregulation represents a novel potential target for treating ALI/ARDS.

## Supplementary Material

Supplementary materials and methods, figures, and tables.

## Figures and Tables

**Table 1:** Sequences of the primers used to quantitate gene expression.

Gene	Forward primer (5′-3′)	Reverse primer (5′-3′)
*Cyp2j9*	AGTCAGTCACCGCCTTTGTG	GTCTCATTGCACGCACTCTC
*Cyp2j6*	GGTGCCCTTGTTGTTAGCAC	GGCTAACAAGGAGCCGGTAG
*Cyp2j5*	TGATGGGTTCATCAGCAGGC	CTTGGCTCATCTGGGTTCCAAT
*Cyp2c29*	CCATGGTTGCAGGTAAACCACAT	TCTGTCCCTGCACCAAAGAG
*Cyp2c44*	CAAGGTACCCCGAGTGAAGAA	CACGGCATCTGTATAGGGCA
*Cox-2*	CATCCCCTTCCTGCGAAGTT	CATGGGAGTTGGGCAGTCAT
*Nox-2*	GACTGCGGAGAGTTTGGAAG	GGTGATGACCACCTTTTGCT
*Tnf-α*	AGCCCCCAGTCTGTATCCTT	CTCCCTTTGCAGAACTCAGG
*Mcp-1*	GTCCCTGTCATGCTTCTGG	GCGTTAACTGCATCTGGCT
*Trem-1*	CTGTGCGTGTTCTTTGTC	CTTCCCGTCTGGTAGTCT
*Nlrp3*	TACGGCCGTCTACGTCTTCT	CGCAGATCACACTCCTCAAA
*pro-caspase-1*	CACAGCTCTGGAGATGGTGA	CTTTCAAGCTTGGGCACTTC
*Asc*	GACAGTACCAGGCAGTTCGT	AGTCCTTGCAGGTCAGGTTC
*pro-Il-1β*	CAGGCAGGCAGTATCACTCA	AGCTCATATGGGTCCGACAG
*Nf-κb/p65*	GGAGGCATGTTCGGTAGTGG	CCCTGCGTTGGATTTCGTG
*β-actin*	TTCCAGCCTTCCTTCTTG	GGAGCCAGAGCA GTAATC

**Table 2:** Antibody sources and dilutions.

Antibodies	Source	Catalog	Dilution ratio
**Primary antibodies for Western blotting**			
Anti-sEH monoclonal antibody	Abcam	ab155280	1: 1000
Anti-COX-2 polyclonal antibody	Proteintech	12375-1-AP	1: 2000
Anti-Nrf2 monoclonal antibody	CST	#12721	1: 1000
Anti-TREM-1 polyclonal antibody	Proteintech	11791-1-Ap	1: 1000
Anti-NLRP3 monoclonal antibody	CST	#15101	1: 2000
Anti-IL-1β polyclonal antibody	R&D	AF-401-NA	1: 2000
Anti-pro-caspase-1/p10/p20 monoclonal antibody	Abcam	Ab179515	1: 1000
Anti-I-κBα monoclonal antibody	Abcam	Ab32518	1:2500
**Secondary antibodies for Western blotting**			
Anti-β-actin polyclonal antibody	SAB	#21338	1: 7500
Anti-β-tubulin monoclonal antibody	Servicebio	#48885	1: 2000
Antibodies for IP assay			
Anti-NLRP3 monoclonal antibody	AdipoGen	AG-20B-0014-C100	1:1000
Anti-ASC monoclonal antibody	CST	67824S	1:1000

## Abbreviations

ALI: acute lung injury
ARA: arachidonic acid
ARDS: acute respiratory distress syndrome
ASC: apoptosis-associated speck-like protein containing a CARD
ATP: adenosine triphosphate
BALF: bronchoalveolar lavage fluid
COX: cyclooxygenase
CYP: cytochrome P450
DHETs: dihydroxyeicosatrienoic acid
DMLs: designing multiple ligands
EETs: epoxyeicosatrienoic acids
H&E: Hematoxylin-Eosin
IP: immunoprecipitation
IL-1β: interleukin-1 beta
LPS: lipopolysaccharide
LOX: lipoxygenase
MCP-1: monocyte chemotactic protein 1
MDA: malondialdehyde
NF-κB: nuclear factor kappa-B
NLRP3: NACHT, LRR and PYD domains-containing protein 3
NOX-2: NADPH oxidases 2
Nrf2: nuclear factor-erythroid-2-related-factor-2
PGs: prostaglandins
PTUPB: 4-(5-phenyl-3-{3-[3-(4-trifluoromethylphenyl)-ureido]-propyl}-pyrazol-1-yl)-benzenesulfonamide
RT-qPCR: real-time quantitative polymerase chain reaction
ROS: reactive oxygen species
sEH: soluble epoxide hydrolase
SOD: superoxide dismutase
TNF-α: tumor necrosis factor-alpha
