# Supplementary material for: A COX-2/sEH dual inhibitor PTUPB alleviates lipopolysaccharide-induced acute lung injury in mice by inhibiting NLRP3 inflammasome activation
Source: Theranostics. 2020 Mar 26;10(11):4749–61. doi: 10.7150/thno.43108 (PMC7163435; doi:10.7150/thno.43108)
Supplement: Supplementary file 1 — Supplementary materials and methods, figures, and tables. [file thnov10p4749s1.pdf]

Materials and Methods

Detection of the gene expressions of *Aloxs*

The gene expression of *Alox5*, *Alox12*, and *Alox15* was detected by RT-qPCR. The sequences of primers are shown in Table S1.

Macrophages depletion *in vivo*

Macrophages were depleted as previous studies [1, 2]. ALI mice randomly received a single dose of liposomal clodronate or liposomal PBS without clodronate (from F70101C-AC-2, FormuMax, USA) *via* the tail vein (150 μL) and tracheal instillation (50 μL) 24 h before the induction of ALI. Clodronate liposomes encapsulated dichloromethylene diphosphonate. The concentration of the liposomal clodronate suspension was 7 mg/mL, which led to macrophage depletion.

Immunofluorescence staining

For the lung tissues, the paraffin-embedded sections (3-μm) of the lung tissue were baked at 65 °C for 2 h. After deparaffinization, antigen retrieval, and serum blocking, the sections were incubated with primary antibodies against F4/80 or MPO at 4 °C overnight. For primary murine peritoneal macrophages, after treatment, cells were washed and fixed with 4% paraformaldehyde for 15 min, permeabilized with 0.1% TritonX100 for 15 min, and blocked with 1% BAS for 30 min, then incubated with primary antibodies against NF-κB/p65 at 4 °C overnight. Then the sections or cells were incubated with the relevant secondary antibody for 1 h at room temperature. The nuclei were stained with fluorescent dye 4',6-diamidino-2-phenylindole (DAPI) for 5 min. All the images were captured on a Nikon ECLIPSE Ti microscope (Nikon, Tokyo, Japan), and the sections and cells were examined at 400× magnification. The antibodies used in the study are shown in Table S2.

Supplementary Tables

Table S1. Sequences of the primers used to quantitate gene expression.

Gene	Forward primer (5′–3′)	Reverse primer (5′–3′)
Alox5	GTTCCCATGTTACCGCTGGA	TACGTCTGTGCTGCTTGAGG
Alox12	TTCTCCGGATCCCTCAACCT	CGGGAACGTCGAAGTCAAAC
Alox15	GTAACCCACCACGTTCAGCA	AAAGCGGAAGCGATCAAGGA

Table S2. The antibodies used Immunofluorescence staining

Antibodies	Source	Catalog	Dilution ratio
F4/80 polyclonal antibody	Servicebio	GB11027	1:500
MPO polyclonal antibody	Servicebio	GB11224	1:500
NF-κBp65 polyclonal antibody	Servicebio	GB11142	1:200
DyLight 488 Conjugated AffiniPure Goat Anti-rabbit IgG (H+L)	Boster	BA1127	1:400
DyLight 550 Conjugated AffiniPure Goat Anti-rabbit IgG (H+L)	Boster	BA1135	1:400

Supplementary Figures and Figure legends

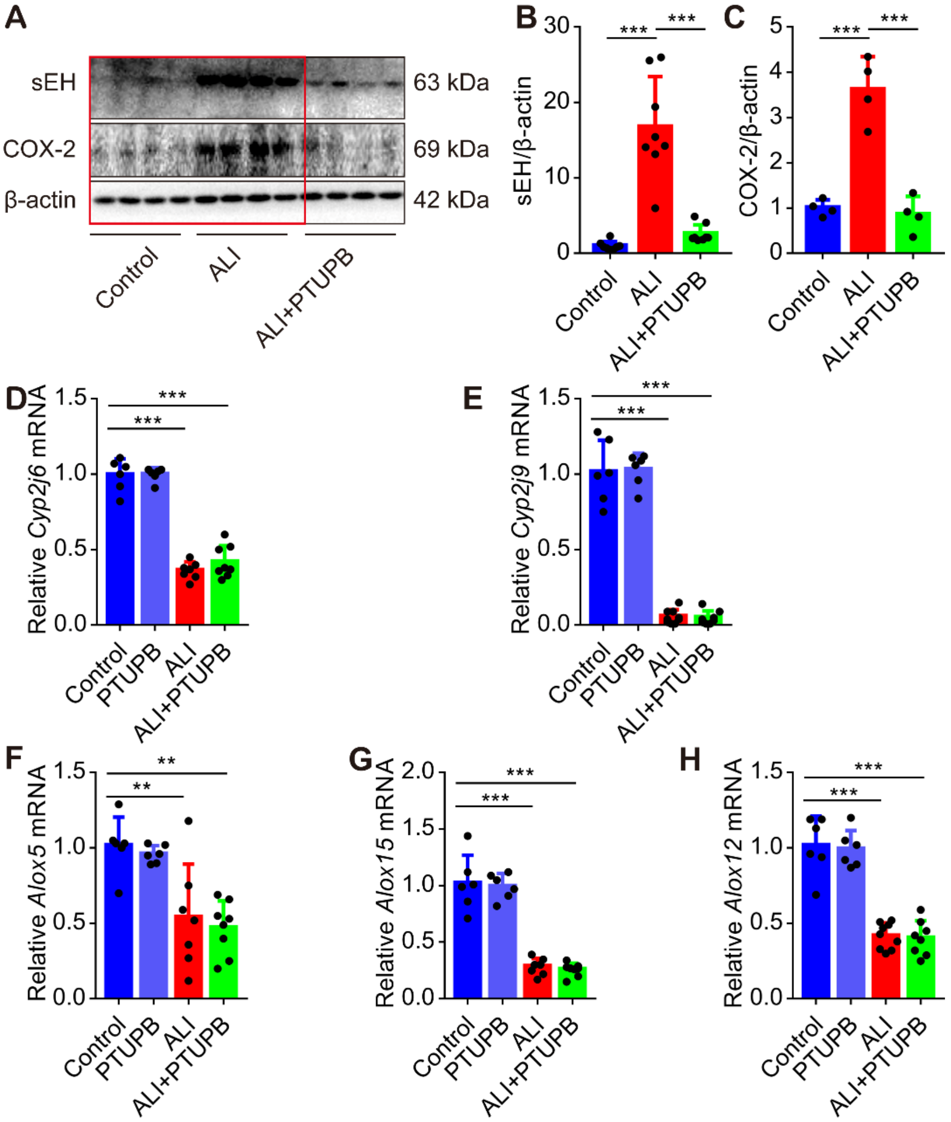

Figure S1. PTUPB restores the expression of sEH and COX-2 rather than *Cyps* and *Aloxs* in the lung of LPS-treated mice with and without PTUPB treatment. C57BL/6 mice were intraperitoneally injected with PTUPB 1 h before the LPS administration. Twelve hours after the LPS administration. Expression of sEH and COX-2 protein in lung tissue was detected by Western blotting (A-C, $n = 4\text{-}8$). Picture in the red frame is also shown in Fig. 1. mRNA expression of *Cyp2j6*, *Cyp2j9*, *Alox5*, *Alox12*, and *Alox15* in the lungs was detected by RT-qPCR (D-H, $n = 6\text{-}8$). Data are expressed as the mean ± SD. ** $P < 0.01$ and *** $P < 0.001$.

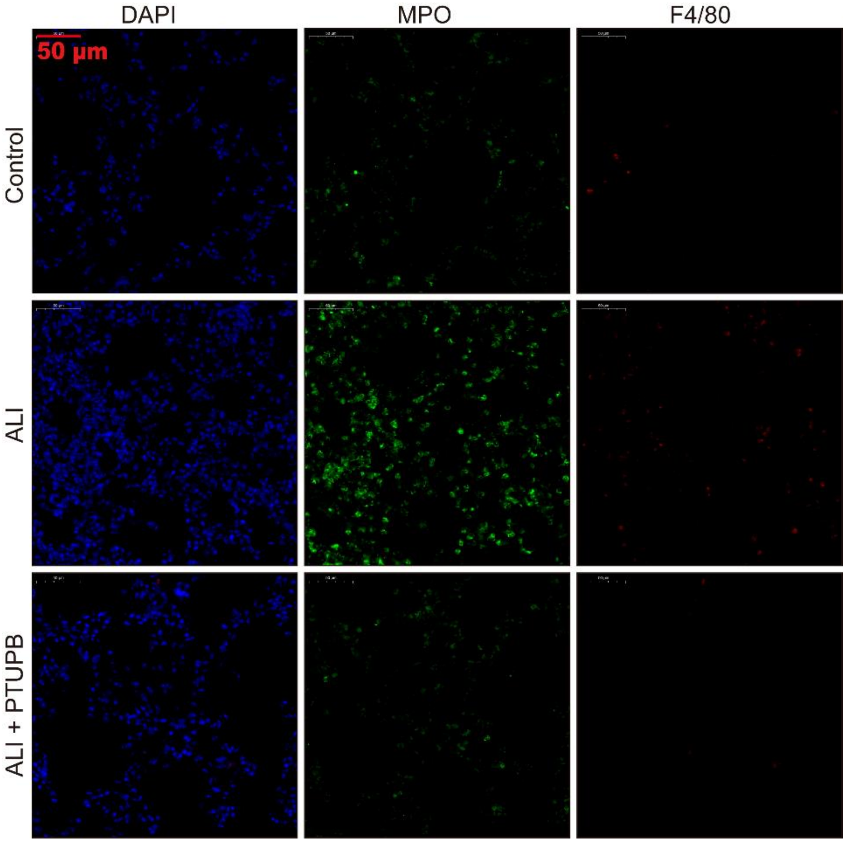

Figure S2. PTUPB reduces the infiltration of the neutrophils and macrophages in LPS-induced lung injury mice. C57BL/6 mice received LPS injection (5 mg/kg, *i.t.*) with or without PTUPB pre-treatment (5 mg/kg) for 1 h. Twelve hours after the LPS injection, immunofluorescent assay was used to detect the expression of MPO (Green) and F4/80 (Red) as the marker of neutrophils and macrophages, respectively.

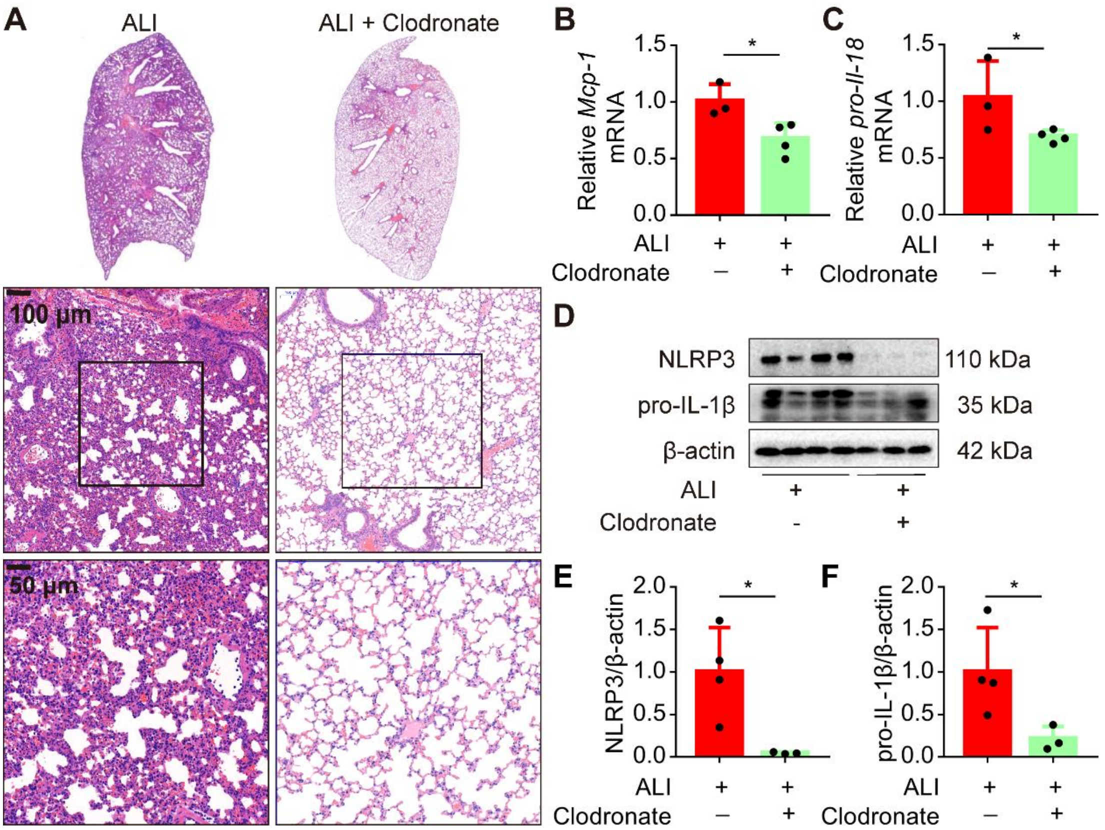

Figure S3. Macrophages depletion ameliorates the lung tissue injury of ALI mice. Liposomal clodronate or liposomal PBS (150 μL, *i.v.* and 50 μL, *i.t*) was administered to mice 24 h before the induction of ALI by LPS (5 mg/kg, *i.t.*). Lung histopathology was performed with H&E staining (A). mRNA expression of *Mcp-1* (B, n = 3-4) and *pro-Il-18* (C, n = 3-4) in the lungs was detected by RT-qPCR. Protein expression of NLRP3 and pro-IL-1β in lung tissue was detected by Western blotting (D-F, n = 3-4). Data are expressed as the mean ± SD. * $P < 0.05$.

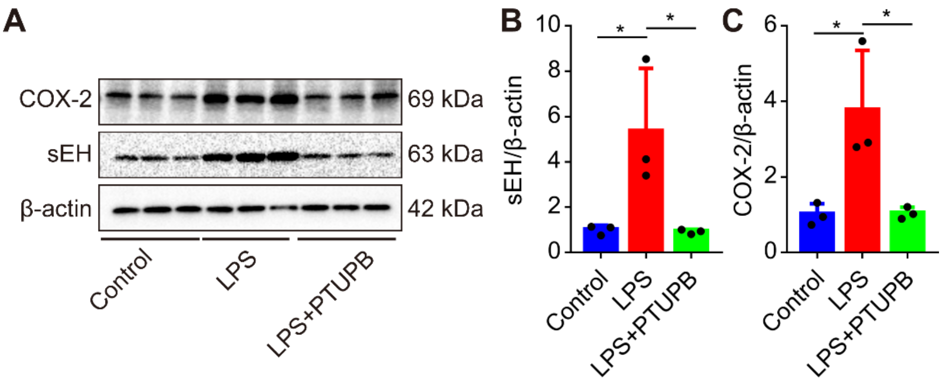

Figure S4. PTUPB restores the dysregulation of sEH/COX-2 in LPS-stimulated primary murine macrophages. Primary murine macrophages received LPS treatment (10 ng/mL) with or without PTUPB pre-treatment (1 μM) for 1 h. Twelve hours after the LPS treatment, protein expression of COX-2 and sEH in primary murine macrophage was detected by Western blotting (A-C, $n = 3$). Data are expressed as the mean ± SD. * $P < 0.05$.

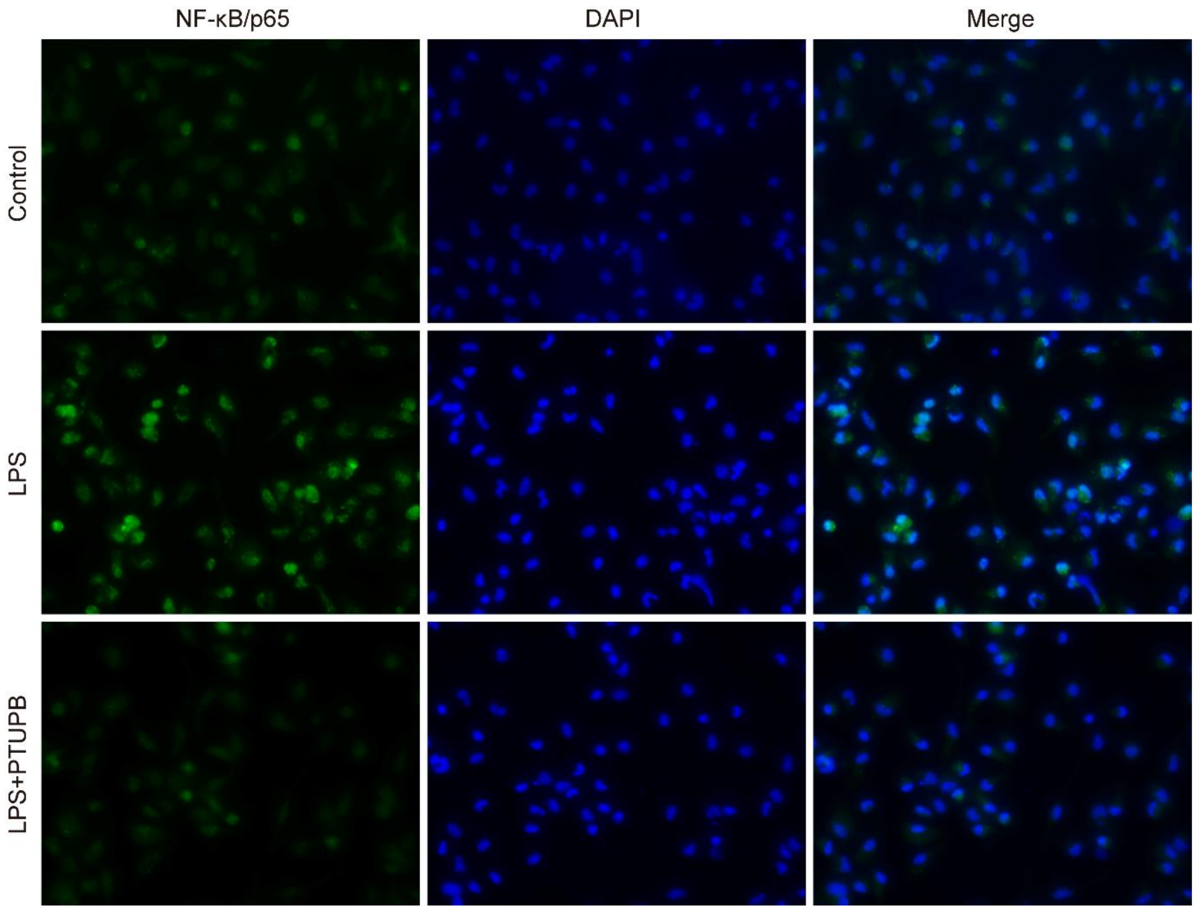

Figure S5. PTUPB inhibits the nuclear translocation of NF-κB/p65 in LPS-stimulated primary murine macrophages. Primary murine macrophages received LPS treatment (10 ng/mL) with or without PTUPB pre-treatment (1 μM) for 1 h. Twelve hours after the LPS treatment, immunofluorescent assay was used to detect the nuclear translocation of NF-κB/p65 (green) and DAPI (blue) as the marker of the nucleus.

References:

1. Moreno SG. Depleting Macrophages In Vivo with Clodronate-Liposomes. Methods Mol Biol. 2018; 1784: 259-62.
2. Frank JA, Wray CM, McAuley DF, Schwendener R, Matthay MA. Alveolar macrophages contribute to alveolar barrier dysfunction in ventilator-induced lung injury. Am J Physiol Lung Cell Mol Physiol. 2006; 291: L1191-8.
